# The Level of Knowledge of Parkinson's Disease among Nonprofessional Caregivers in a Movement Disorders Center in Turkey

**DOI:** 10.1155/2017/2652361

**Published:** 2017-03-01

**Authors:** Murat Gultekin, Ayse Caglar Sarılar, Ayten Ekinci, Gozde Erturk, Meral Mirza

**Affiliations:** ^1^Department of Neurology, Erciyes University School of Medicine, Kayseri, Turkey; ^2^Department of Biostatistics, Erciyes University School of Medicine, Kayseri, Turkey

## Abstract

*Introduction*. Only a few studies have been conducted to determine the level of knowledge among caregivers about Parkinson's disease (PD). The aim of the current study was to determine the knowledge of PD among caregivers at a movement disorder clinic in Turkey.* Methods*. We conducted a questionnaire based interview with the subjects in a tertiary care neurology facility in Turkey. The questions were divided into two parts covering the symptomatology and treatment of PD. A questionnaire consisting of 10 questions was applied to the subjects who had to mark the correct option in a stipulated time.* Results*. Eighty caregivers were included in the study. The caregivers' mean age was 47.94 years (SD = 12.40). There were 47 female caregivers (58.8%). The most well-known question was that the number of drugs given to the patient may vary with time (76.3%), whereas “the benefit noted in the patient's treatment decreases over time” was the least known question (11.3%).* Discussion*. This study is the first in our country and shows the necessity to increase the knowledge of PD among caregivers and the public. Education programs may have a positive role in imparting knowledge to the caregivers of PD patients.

## 1. Introduction

Parkinson's disease (PD) is a progressive neurodegenerative illness [1]. In the diverse treatment of chronic diseases, the training of the caregiver has an important role in the success of treatment [2, 3]. In progressive PD, in many areas such as the time when medicine is taken, determination of the duration of medicines, observation of the response to treatment, and monitoring the motor complications related to the disease and treatment, the contribution of the caregiver affects the patient directly.

There are very few studies about caregivers' level of information about PD [4]. In these studies, the caregivers' level of information about PD was found to be very low [5, 6]. Most caregivers mention that following the diagnosis of their relatives they have insufficient information [7]. The lack of general information of both patients with PD and their caregivers may cause communication problems among the doctor, patient, and the caregiver. This situation negatively affects the treatment of the disease and the daily life activities of the patient directly. There are no data on this subject in the literature in our country.

The purpose of this study is to measure the knowledge and attitude of caregivers of PD patients about Parkinson's disease. In this way, during the informing and training of caregivers of PD patients, what is significant and remarkable will be revealed.

## 2. Methods

### 2.1. Sample

The study included 80 volunteer caregivers of PD patients who were diagnosed according to the criteria of the United Kingdom Parkinson's Disease Brain Bank and followed up in Erciyes University Medical Faculty, at the Department of Neurology movement disorder clinic between January 2016 and April 2016. The exclusion criterion was determined as caregivers under 18 years of age. This study was approved by the Ethics Committee of Clinical Research of Erciyes University on 18.12.2015 (with number 538). The data were obtained after giving information about the purpose of the study and with the written consent of the participants. The participants first of all filled in the demographic information form. Next, to determine the level of information of the caregivers about PD, a questionnaire was applied. The necessary information about the clinical status of the patient for the study was added by a neurologist with experience in movement disorders. The questions were answered by the participant caregivers by marking the correct answer in accordance with their own knowledge.

### 2.2. The Evaluation Tools Used

#### 2.2.1. Demographic Information Form

In keeping with the purpose of the study, a form was applied by the researchers to the caregivers and patients; it meets the inclusion criteria of the study to determine information on age, gender, level of education, and other demographic information. With the current form, besides the demographic information, the degree of relationship of the caregivers to the patient, caregiving situation, the duration of PD, and the clinical staging information according to the of Hoehn-Yahr scale were asked and recorded.

#### 2.2.2. Questionnaire Form

A questionnaire was applied to the caregivers of the PD patients to determine their information level about the treatment of disease. The questionnaire has ten questions of which eight are multiple choices and two are open ended. The questions were designed by the researchers after an analysis of the general literature.

### 2.3. Statistical Analysis

For the comparison of the categorical data in the study, the exact method of Pearson chi-square test statistics was used. In the comparison of two groups, in independent samples, the Student *t*-test was used. In the comparison of more than two groups, one-way variance analysis (ANOVA) was used. In multiple comparison tests (post hoc) Tukey HSD test statistics was used. The evaluation of the data distribution was checked with Shapiro-Wilks test statistics. The appropriate test statistics have been chosen and test statistic was made. The analyses of the data have been evaluated with SPSS 22 (IBM Corp; Armonk, NY, USA). The significance level has been accepted as* p* > 0.05.

## 3. Results

The study included 80 caregivers of PD patients. Forty-seven of these were female (58.8%) and the mean age of the caregivers was found as 47.94 years (SD = 12.40). It was observed that most of the caregivers were the spouses of the patients (43.8%) or their children (43.8%). Also 67.6% of the caregivers were retired or not working. It was also found that only 18.8% of the caregivers were university graduates. The mean duration of caregiving was 7.8 years and it was determined that most of them (67.5%) cared for the patients full time. At the same time, it was also determined that most of the caregivers (65%) did not have previous experience of caregiving. The demographic characteristics of the caregivers and patients with PD are summarized in Table 1.

Forty-seven (58.8%) of the patients were female. Nineteen (23.8%) of the patients were illiterate. The mean duration of the disease was found as 6.96 years (SD = 5.69). Of the patients, 53.7% said that they did not do any exercises. In addition, 21.2% of the patients said that they did not go for regular clinical checkups. Also, we found that there was no significant difference between the knowledge level and caregiving situation (*p* = 0.339).

The answer rates of the caregivers for the first 8 multiple choice questions in the questionnaire are shown in Table 2. Regarding this, the areas in which caregivers had the least information about PD are as follows: 11.3% of the caregivers answered that the current treatment is aimed at controlling the symptoms of the disease; 13.8% of the caregivers answered that the benefit of treatment can change over time; 16.3% of the caregivers answered that they only knew of oral treatment for PD. In addition, the question to which the caregivers answered as “I don't have an idea” is related to nonoral treatments for PD (52.5%). The best known treatment option is the possibility of changing the number of drugs used by the patient (76.3%).

With the correct answers to the questions, no significant difference was found among the stage of the disease, duration of the disease, and the level of education of caregivers. However, when the genders of the caregivers were checked, for only the first question (What is your opinion about the state of Parkinson disease?) was there a significant difference between the answers (*p* = 0.023). In this question, the progressive nature of Parkinson's disease was asked. Thirteen men answered correctly (30.2%) whereas 30 women (69.8%) answered correctly.

The last two questions were open ended. To the 9th question, namely, “How often should a patient with PD come to clinical checks?,” 56.3% of the caregivers answered that patients should go to clinical checks at two-month periods and 21.2% of them said at four-month periods. However, 18.7% of the caregivers answered that patients should go to clinical checks if they have any complaints related to PD. This question was asked to determine patients who receive inadequate treatment and do not come to the clinic.

In the 10th question, the caregivers were asked to guess about the complaints of the patients, namely, “Could they guess that patients had PD before it was diagnosed?” According to this, before diagnosis, only 15% of the caregivers mentioned that their patients could have PD.

In addition to the questionnaire, caregivers were asked about their sources for information. Most of them named “doctor only” (61,3%) as their source. The Internet was referred to by 10%; other carers were referred to by 6.3% and books-brochures were referred to by 5%.

## 4. Discussion

In this study, we present our country's first data concerning the level of knowledge and perceptions of caregivers regarding PD. According to these data, it was determined that 65% of caregivers are not experienced or trained in providing care to PD patients or to those with a chronic disease. However, in spite of the progress in neurodegenerative diseases like PD, it was shown that caregivers do not have sufficient information about PD. Therefore, it is obvious that the patient treatment may be negatively affected. Our findings show a similarity with other studies on this subject [4, 8].

In a study conducted by Yadav et al. in India a multiple choice questionnaire comprising 10 questions was applied to caregivers. It was shown that participants are misinformed mostly in the following areas: biochemical abnormality in PD, stage in which patients are considered suitable for deep brain stimulation and the surgical treatment methods available in PD. In these matters, it was shown that caregivers are misinformed regarding the surgical methods available in PD [4]. In our data, the fact that caregivers believe that drugs can totally cure PD (78.8%) was the most widely found misunderstanding. This was followed, respectively, by the beliefs that the benefit of treatment will not change over time (47.5%) and that there are no equipment supported treatments for PD (31.3%).

There are different studies about caregivers' level of information about PD in the literature. Tan et al. conducted one study to show comparing knowledge of genetic testing in PD patients and caregivers in American and Asian population [5]. It was found that American PD patients had a higher level of knowledge of PD genetics than Asian PD patients. A greater number of American PD patients and caregivers reported a positive attitude towards the potential medical benefits of genetic testing compared to their Asian counterparts. Furthermore, Sakanaka et al. conducted a similar study to show knowledge of genetic PD patients and caregivers [6]. It was shown that only 51.6% of PD participants and 55.6% of caregivers knew that “scientists have identified genes associated with a higher risk of developing PD.” Also, it was concluded that PD patients may benefit from education and genetic counseling on the implications of genetic testing.

For optimum daily living activities (DLA), patients with PD and their caregivers need to know about the disease and its treatment. In particular, this situation is essential in the middle and advanced stages of PD [9]. For example, some patients in this period may need nonoral treatments because of the limitation of their DLA due to motor complications. In fact, with equipment supported treatments, patients with PD and their caregivers' DLA can be significantly improved [10]. However, our data revealed that few caregivers (16.3%) have information about these treatments.

Although the caregivers in our working group were not trained, we observed that they answered correctly the questions about when medicines should be taken and the changes in the number of medicines. This is thought to be due to the patients' caregivers being well-informed (73.8%) and is thought to be linked to interviews with experts in the field. This situation may depend on interviews between caregivers and doctors in clinics and later may depend on the questionnaire. In our working group, the knowledge resource of PD is mostly “doctors” (61.3%) which verifies this situation.

More information about PD in printed and electronic media is needed and the establishment of clinics dedicated to movement disorders and centers of excellence with special education programs would go a long way in increasing the awareness and knowledge of PD among caregivers in our country. Furthermore, the practitioners should give sufficient time to caregivers and help in providing them with correct information by means of books, brochures, and websites.

*Limitations of the Study*. Because the number of caregivers is small and ours is a small scaled and local study, it may not reflect our data very strongly. Most of the questions in the questionnaire including subjects' knowledge about treatment reveal only one side of PD.

*Strengths of the Study*. Although our study is small scaled, because it reflects the first data in our country, it is considered as important. The information presented herein may help to fill the gap in caregivers' training.

## 5. Conclusion

Patient and caregiver education programs may have a positive role in imparting knowledge to PD patients and their caregivers. Studies are needed to understand the knowledge, attitude, and perceptions regarding Parkinson's disease among the general population.

## Supplementary Material

Questionnarie: There is ten questions about PD, one about epidemiology, three on symptoms and seven on treatment. Correct answers are in bold letters.

## Figures and Tables

**Table 1:** Demographic characteristics of the participants (caregivers and patients).

*n* = 80	Caregiver		Patients with PD	
	*n*	*%*	*n*	*%*
Gender				
Female	47	58.8	39	48.8
Male	33	41.3	41	51.3
Level of education				
Illiterate	4	5.0	19	23.8
Literate/elementary	39	48.8	41	51.3
Middle/high	22	27.6	14	17.5
University	15	18.8	6	7.5
Occupation				
Not working	39	48.8	40	50
Retired	15	18.8	40	50
Working	26	32.5		
Caregiving situation				
Rarely	14	17.5		
Mostly	12	15		
Full time	54	67.5		

Age [mean (SD)]	47.94 (12.40)		62.46 (10.19)	
Duration of illness [mean (SD)]	—		6.96 (5.69)	

**Table 2:** The frequencies of the answers in the questionnaire (*n* = 80).

Questions	Correct answer (%)	Wrong answer (%)	No idea (%)
(1) What is the state of Parkinson disease?	43 (53.8)	31 (38.8)	6 (7.5)
(2) Does the number of drugs change during treatment?	61 (76.3)	9 (11.3)	10 (12.5)
(3) Are there nonmotor symptoms in Parkinson disease?	44 (55)	27 (33.8)	9 (11.3)
(4) *Are there treatment choices other than oral? *	13 (16.3)	25 (31.3)	42 (52.5)
(5) *What is the purpose of the current treatment in Parkinson disease?*	9 (11.3)	63 (78.8)	8 (10)
(6) What is the effect of exercise in Parkinson disease?	54 (67.5)	7 (8.8)	19 (23.8)
(7) How often should the drugs be taken in Parkinson disease?	57 (71.3)	21 (26.3)	2 (2.5)
(8) *Does the benefit to the patient change over time?*	11 (13.8)	38 (47.5)	31 (38.8)

Lower frequencies for the correct answers are shown in darker print.
