# Supplementary material for: The Level of Knowledge of Parkinson's Disease among Nonprofessional Caregivers in a Movement Disorders Center in Turkey
Source: Parkinsons Dis. 2017 Mar 1;2017:2652361. doi: 10.1155/2017/2652361 (PMC5350480; doi:10.1155/2017/2652361)
Supplement: Supplementary file 1 — Questionnarie: There is ten questions about PD, one about epidemiology, three on symptoms and seven on treatment. Correct answers are in bold letters. [file 2652361.f1.pdf]

Annex-1: Questionnaire

1 What is your opinion about the state of Parkinson Disease ?

a) Temporary b) Permanent c) Progressive d) Don’t have an idea

2 Can the number of the drugs which your patient take change in time ?

a) Yes

b) No

c) No idea

3 Apart from the movement disorder, are there any symptoms originate from the disease ?

a) Yes (Please mention)…………………………………………………………

b) No

c) No idea

4 Are there any other treatment choices except from oral treatment (medicine) in the treatment of Parkinson Disease ?

a) Yes (Please mention) …………………………………………………………………

b) No

c) No idea

5 What is the purpose of the current medical treatment ?

a) To reduce the symptoms of disease

b) Healing of the disease in time completely

c) To stop the development of disease in time

d) I don’t have an idea

6 What do you think about exercising in parkinson Disease ?

a) The disease becomes worse.

b) Nothing changes

c) Relaxes the body and develop the movements

d) I don’t have an idea

7 How often do the medicines taken in Parkinson Disease ?

a) Morning and evening

b) Morning, noon and evening

c) At specified hours in the day

d) I don’t have an idea

8 How can the benefit of your patient becomes in time ?

a) The effect of the treatment stays costant

b) The effect of the treatment increases in time

c) The effect of the treatment decreases in time

d) I don’t have an idea

9 What do you think about the frequency of your patient’s coming for doctor visit ?
………………………………………………………………………..

10 Before it is diagnosed by the doctors, did you guess Parkinson disease ?
Yes (from which symptom ?)……………………………………….
